# Supplementary material for: Patient-reported outcome measures for acne: a mixed-methods validation study (acne PROMs)
Source: BMJ Open. 2021 Mar 19;11(3):e034047. doi: 10.1136/bmjopen-2019-034047 (PMC7986881; doi:10.1136/bmjopen-2019-034047)
Supplement: Supplementary data [file bmjopen-2019-034047supp001.pdf]

Patient Reported Outcome Measures for Acne: a Mixed Methods Validation Study. Supplementary Tables

Supplementary Table 1. Internal consistency findings – Cronbach's alpha

Outcome measure	Cronbach's alpha
Skindex-16	
– Symptoms	0.86
– Emotions	0.95
– Functioning	0.95
– Total	0.96
Compaq	
– Symptoms	0.80
– Social (judgements by others)	0.95
– Social interactions	0.93
– Psychological/Emotional	0.94
– Treatment concerns	0.74
– Total	0.95

Supplementary Table 2. Reliability findings – ICC comparing baseline to 24/48 hours later (n=204)

Outcome measure	ICC (95% CI)
Skindex-16	
– Symptoms	0.96 (0.95, 0.97)
– Emotions	0.97 (0.95, 0.98)
– Functioning	0.96 (0.95, 0.97)
– Total	0.96 (0.94, 0.97)
Compaq	
– Symptoms	0.91 (0.88, 0.93)
– Social (judgements by others)	0.93 (0.91, 0.95)
– Social interactions	0.94 (0.92, 0.95)
– Psychological/Emotional	0.93 (0.91, 0.95)
– Treatment concerns	0.88 (0.84, 0.91)
– Total	0.93 (0.91, 0.95)

Supplementary Information 1. Responsiveness to Change

Correlations were in line with hypotheses for most subscales (see Supplementary Table 3). The Acne-Qol showed the highest correlation with self-reported change and the CompAQ the lowest. The Skindex emotional subscale had the highest correlation with the participants' assessment of change. Using an AUROC approach gave similar results (see Supplementary Table 3).

As expected, the correlations were higher in those who reported a change in treatment during the 6 week period, with all scales above the threshold of 0.70 set for the AUROC on those reporting a change, on all domains apart from the Treatment Concerns and Social domains on the CompAQ. However, there were only 44 such participants and so it is difficult to draw conclusions from this limited sample.

Supplementary Table 3. Responsiveness to change over a 6 week period

Outcome measure	Correlation with self-reported	Correlation with self-reported	AUROC for all	AUROC for those who

	change - all participants (n=167)	change – those who reported a change in treatment during the 6 weeks (n=44)	participants (n=167)	reported a change (n=44)
Acne QoL				
– Symptoms	0.39	0.42	0.72	0.77
– Role Emotional	0.39	0.46	0.72	0.73
– Role Social	0.39	0.38	0.74	0.72
– Self Perception	0.42	0.49	0.72	0.80
Skindex-16				
– Symptoms	0.34	0.37	0.68	0.72
– Emotions	0.43	0.56	0.70	0.72
– Functioning	0.38	0.35	0.70	0.71
– Total	0.47	0.54	0.75	0.77
CompAQ				
– Symptoms	0.28	0.34	0.64	0.71
– Social (judgements by others)	0.31	0.25	0.68	0.65
– Social interactions	0.32	0.34	0.68	0.71
– Psychological/Emotional	0.40	0.39	0.71	0.76
– Treatment concerns	0.30	0.30	0.64	0.67
– Total	0.43	0.44	0.73	0.76

Supplementary Information 2. Interpretability Findings

There were floor effects for some subscales for both Skindex and CompAQ. Fourteen percent and 16.2% at baseline and 24 hours, respectively, scored 0 on the functioning subscale within the Skindex. Similarly, for the CompAQ, a high number of participants scored 0 for the social judgement and social interactions subscales at baseline and 24 hours; 11% and 18.9% for the social judgement scale and 16.4% and 24.48% for the social interactions subscale. These floor effects may be due to the number of participants who reported that their acne was clear or almost clear
